# The Content of Phenolic Compounds in Leaf Tissues of White (*Aesculus hippocastanum* L.) and Red Horse Chestnut (*Aesculus carea* H.) Colonized by the Horse Chestnut Leaf Miner (*Cameraria ohridella* Deschka & Dimić)

**DOI:** 10.3390/molecules190914625

**Published:** 2014-09-15

**Authors:** Jan Oszmiański, Stanisław Kalisz, Wojdyło Aneta

**Affiliations:** 1 Department of Fruit, Vegetable and Grain Technology, Wroclaw University of Environmental and Life Sciences, 37/41 Chełmońskiego St., 51630 Wroclaw, Poland; E-Mail: aneta.wojdylo@up.wroc.pl; 2 Division of Fruits and Vegetables Technology, Department of Food Technology, Warsaw University of Life Sciences (SGGW), Nowoursynowska 166, 02-787 Warsaw, Poland; E-Mail: Stanislaw_kalisz@sggw.pl

**Keywords:** horse chestnut, polyphenols, *Cameraria ohridella*, LC-MS QTof

## Abstract

Normally, plant phenolics are secondary metabolites involved in the defense mechanisms of plants against fungal pathogens. Therefore, in this study we attempted to quantify and characterize phenolic compounds in leaves of white and red horse chestnut with leaf miner larvae before and after *Cameraria ohridella* attack. A total of 17 phenolic compounds belonging to the hydroxycinnamic acid, flavan-3-ols and flavonol groups were identified and quantified in white and red horse chestnut leaf extracts. Significantly decreased concentrations of some phenolic compounds, especially of flavan-3-ols, were observed in infected leaves compared to the non-infected ones. Additionally, a higher content of polyphenolic compounds especially (−)-epicatechin and procyanidins in leaves of red-flowering than in white-flowering horse chestnut may explain their greater resistance to *C. ohridella* insects.

## 1. Introduction

*Aesculus hippocastanum* L. *(Hippocastanaceae)* is a large tree with sessile leaflets, commonly known as horse chestnut, a rapidly-growing tree that can reach a height of 36 meters. Flowers are white or pink with a small red spot and prickly fruits. It is widely cultivated as an ornamental tree in the mountains of the Balkans in southeastern Europe. The white-flowering horse chestnut tree (*Aesculus hippocastanum* L.) represents one of the most commonest ornamental trees found within European towns, cities, parks and woodlands. Over the past two decades, however, this particular tree species has suffered heavily from attacks by a leaf mining insect known as *Cameraria ohridella* Deschka & Dimić, the horse chestnut leaf-miner. It was discovered for the first time in Macedonia in the vicinity of lake Ohrid in 1985. The second time this species was described as a *species novum—C. ohridella—*in 1986 and to date it is the only representative of this genus in Europe [1,2].

Since its detection in Macedonia, *C. ohridella* has migrated to the north, western and central part of Europe [3]. Baraniak, Walczak and Zduniak [4] described that damage to the plant is caused by the larvae living inside leaves and feeding on the sap and palisade parenchyma. Pupation takes place inside the leaf. The leaf miner grows quickly and the female lays 20–40 eggs; there are three generations per year in Central Europe. The number of generations depends on food sources and weather conditions and climatic [4]. In the climate conditions of Central Europe imagoes of the first generation appear in May, of the second in June, and of the third in September [5]. Foliar damage occurs when larva tunnel into the leaves of the tree, causing physical destruction of leaf tissue [6]. Infected leaves are covered in small brown patches which spread rapidly across the entire canopy, giving the tree an autumnal appearance [7]. Eventually leaves die and fall prematurely. If new leaves develop they can be re-infected [8,9]. The appearance and mass occurrence of the horse chestnut leaf miner (*C. ohridella*) has had a negative effect on decorativeness of the attacked trees. This weakened condition of the infested trees makes them more susceptible to other enemies and diseases, which would not be harmful in normal circumstances. *C. ohridella* attacks the white-flowering chestnut tree (*Aesculus hippocastanum* L.), more often than red horse chestnut (*Aesculus carea* H.) The red horse chestnut is rarely considered a host of *C. ohridella* because despite the abundant oviposition by females of each generation, the larvae usually die within leaf tissues before they reach the third stage [10]. However, Dzięgielewska *et al.* [11] found that the horse chestnut leaf miner is able to develop two full generations on the red horse chestnut under special circumstances: heavy infestation of white horse chestnut in the vicinity, high *C. ohridella* population numbers and mild winters in several consecutive years at the location. Nevertheless, the number of larvae and the leaf damage on the red horse-chestnut are relatively low, *i.e.*, a maximum 10% of the leaves is damaged throughout the vegetative period [11].

Plants develop defense mechanisms which are aided by some biochemical molecules. Unlike animals, plants cannot synthesize defense antibodies, but can produce numerous antimicrobial substances called phytoalexins. Many of the phytoalexins or preformed chemicals belong to the phenolics group [12], which being toxic to pathogens, are involved in the natural defense reactions of plant against various diseases. These compounds are produced and accumulated at a higher rate after infection [13,14]. 

The presence of phenolic compounds in *A. hippocastanum* had been reported in the literature [15]. The antioxidant activity of the seeds was attributed to the presence of flavonoids, as indicated in the report of Kapusta *et al.* [16]. Hübner *et al.* [17] demonstrated that seeds of *A. hippocastanum* contained glycoside and acylated forms of quercetin and kaempferol. Additionally, the leaves revealed a high content of polyphenolics, but were relatively poor in antioxidant capacity. The production of larger amounts of tannins is one of the strategies of physiological defense against phytophagous insects. Tannins are derivatives of phenolic compounds with a bitter taste and toxic properties that can discourage or deter animals from feeding. The presence of catechin tannins has been found in the tissues of common horse chestnut [18]. The poor activity of the physiological defense strategy, associated with the presence of tannins in the horse chestnut leaves, can be attributable to the fact that the larvae do not feed on avoid the tannin-containing tissues, e.g. the epidermis. Tomczyk *et al.* [19] hypothesized that glycosides produced by the white horse chestnut are not seriously dangerous to *C. ohridella*. 

However, little is known of the variability of phenolic compounds in the leaves of red and white horse chestnut despite the fact that a thorough understanding of the variability in leaf phenolic composition is considered a prerequisite for understanding their role in defense mechanisms against leaf miner larvae. Up to now no information has been obtained on the accumulation of phenolic compounds during the period when horse chestnut was attacked by the leaf mining insect. Therefore the aim of this study was to identify and quantify the main phenolic compounds in leaves of white and red horse chestnut with leaf miner larvae before and after *C. ohridella* attack. 

## 2. Results and Discussion 

### 2.1. Identification of Phenolics in Horse Chestnut Leaves

**Figure 1:**
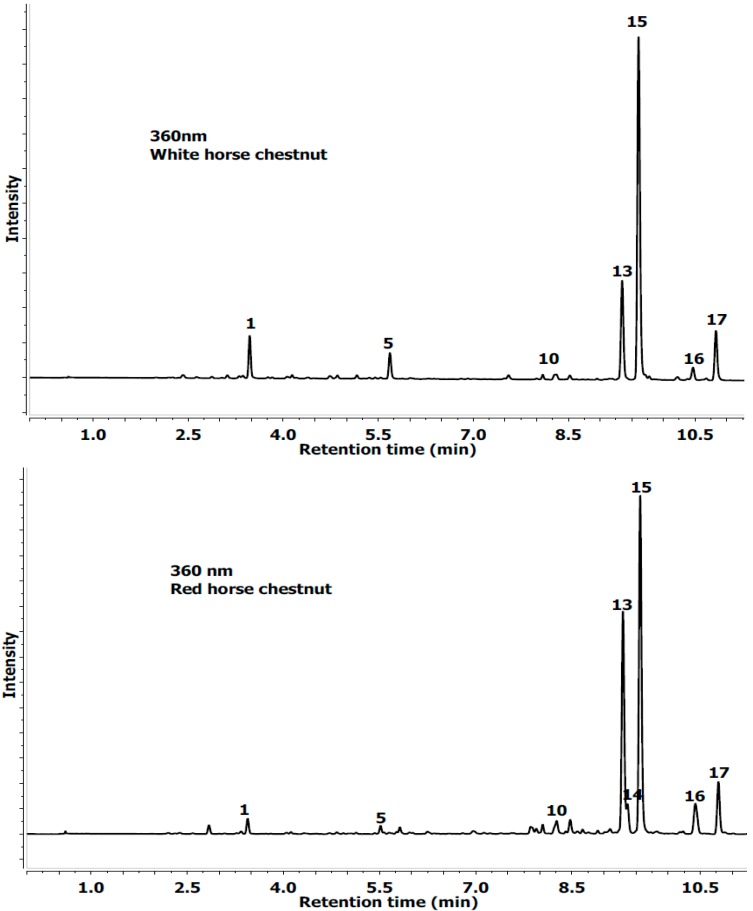
UPLC chromatogram profile of white and red horse chestnut leaves extracts at 360 nm (phenolic acid and quercetin derivative). Abbreviations for peak labels see Table 1.

**Figure 2:**
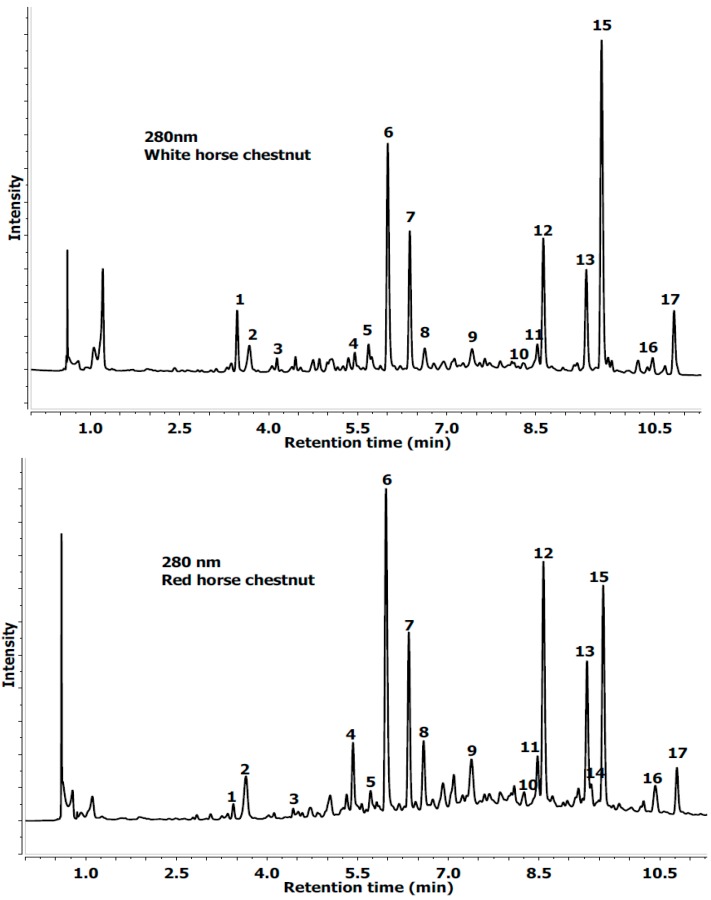
UPLC chromatogram profile of white and red horse chestnut leaves extracts at 280 nm (flavonols). Abbreviations for peak labels see Table 1.

The extracts from white and red horse chestnut leaves suffering from the attack by a leaf mining insect (*C. ohridella*) were analyzed by a LC-ESI-MS/MS system. Results of qualitative analysis obtained by LC-PDA-MS/MS methods and quantitative analysis obtained by UPLC-PDA are summarized in Table 1 and Table 2 and Figure 1 and Figure 2. A total of 17 phenolic compounds found in white and red horse chestnut leaf extracts were identified and are presented.

Three hydroxycinnamates were detected, one of them, neochlorogenic acid, was identified by comparison with authentic standards. This compound had [M−H]^−^ at *m/z* 353 and fragmentation at *m/z* 191 corresponding to quinic acid and λ_max_ 324 nm was identified as 5-*O*-caffeoylquinic acid. 

Caffeoyl-hexose-rhamnose (compound **3**) and trihydroxycinnamoylquinic acid isomers (compound **5**) were found. They had [M−H]^−^ at *m/z* 487 and λ_max_ 311 nm, as well as [M−H]^−^ at *m/z* 369 and λ_max_ 327 nm, respectively. In the MS-MS analysis of compounds **3** and **5**, fragmentation ions were observed at *m/z* 163 and *m/z* 189, which confirmed caffeic acid and trihydroxycinnamic acid as aglycones, respectively.

Eight flavan-3-ols were detected in leaf extracts: (−)-epicatechin, five A-type procyanidins (two trimers and three tetramers) and two B-type procyanidin dimers (Table 1). Compounds with retention time at 5.45 min and 6.01 min and λ_max_ at 280 nm were identified as procyanidin B2 and (−)-epicatechin. Their retention times, *m/z* and λ_max_ were compared with standards. The second procyanidin B dimer was identified in chestnut horse leaf extracts as well. This compound had the same ion *m/z* 577, fragmentation ions *m/z* 289 and λ_max_ at 280 nm as a procyanidin B2 standard.

**Table 1:** Identification of phenolic compounds in white and red chestnut horse leaves using their spectral characteristic with UPLC-PDA (retention time, λ_max_) and negative ions with LC-ESI-MS.

No Peak	Compounds	R_t_ (min)	λ_max_ (nm)	[MS]^−^ *m/z*	[MS/MS]^−^ *m/z*
1	Neochlorogenic acid	3.47	324	353	191
2	A-type PA-tetramer	3.68	280	1151	863
3	Caffeoyl-hexose-rhamnose	4.16	311	487	163
4	Procyanidin B2	5.45	280	577	289
5	Trihydroxycinnamoylquinic acid isomers	5.68	327	369	189
6	(−)-Epicatechin	6.01	280	289	245
7	A-type PA-trimer	6.36	277	863	575
8	A-type PA-tetramer	6.63	277	1151	863
9	A-type PA-trimer	7.43	277	863	575
10	Quercetin-3-*O*-galacoside	8.33	355	463	301
11	Procyanidin B type dimer	8.54	276	577	289
12	A-type PA-tetramer	8.63	277	1151	575
13	Quercetin-3-*O*-arabinoside	9.35	350	433	301
14	Quercetin-3-*O*-rutinoside	9.40	350	609	301
15	Quercetin-3-*O*-rhamnoside	9.61	347	447	301
16	Keampferol-3-*O*-arabinoside	10.47	340	417	285
17	Keampferol-3-*O*-rhamnoside	10.83	340	431	285

**Table 2:** The content [mg/g dm] of phenolic compounds of the extracts of white and red chestnut horse leaves before and after infection

Compounds	White Horse Chestnut			Red Horse Chestnut	
	June	June	September	September	September
	No Infected Leaves (A)	Infected Leaves (B)	Infected Leaves (C)	No Infected Leaves (D)	Infected Leaves (E)
Neochlorogenic acid	1.26 ǂ	0.44	0.03	0.35	0.19
A-type PA-tetramer	1.10	0.93	0.13	2.04	1.40
Caffeoyl–hexose-rhamnose	0.21	0.06	0.01	0.12	0.06
Procyanidin B2	0.65	0.48	0.08	2.66	0.87
Trihydroxycinnamoylquinic acid isomers	0.64	0.20	0.05	0.05	0.03
(−)-Epicatechin	9.50	7.77	0.86	15.36	3.74
A-type PA-trimer	3.49	2.82	0.32	4.95	4.05
A-type PA-tetramer	0.80	0.54	0.15	2.13	1.32
A-type PA-trimer	0.75	0.81	0.09	1.81	1.16
Quercetin-3-*O*-galacoside	0.20	0.13	0.01	0.30	0.18
Procyanidin B type dimer	0.79	0.74	0.04	1.71	0.39
A-type PA-tetramer	3.66	3.15	0.31	7.86	5.35
Quercetin-3-*O*-arabinoside	2.48	1.66	0.32	4.27	2.86
Quercetin-3-*O*-rutinoside	0.00	0.00	0.00	0.59	0.79
Quercetin-3-*O*-rhamnoside	8.22	5.26	0.79	5.93	4.96
Keampferol-3-*O*-arabinoside	0.39	0.27	0.05	0.89	0.88
Keampferol-3-*O*-rhamnoside	1.55	0.93	0.17	1.19	1.15
Procyanidin polymers	24.40	20.40	2.29	46.38	23.62
**Total **	**60.09**	**46.57**	**5.70**	**90.84**	**52.98**

ǂ mean values.

The A-type procyanidins, trimer and tetramer showed UV-spectrum λ_max_ = 277 nm, trimer ions at *m/z* 863 and tetramer at *m/z* 1151, respectively. These flavanes, (−)-epicatechin and its proanthocyanidin type A have been reported in *A. hippocastanum*. These compounds are primarily found in the bark, leaf buds and fruit pericarp [20]. 

Six flavonols were detected in the chestnut horse leaf extracts: four quercetin derivatives, and two kaempferol derivatives. Quercetin derivatives were represented by: quercetin-3-*O*-rhamnoglucoside (rutinoside), 3-*O*-galactoside, 3-*O*-arabinoside and 3-*O-*rhamonside*.* Standards of quercetin-3-*O*-galactoside, 3-*O-*rutinoside and quercetin-3-*O*-rhamnoside were used for more detailed identification of the sugar units of quercetin glycosides.

Peaks with R_t_ = 8.33, 9.40 and 9.61 min, had λ_max_ values of 355 and 350 nm, respectively. All compounds had a typical quercetin ion fragment at *m/z* 301. Two derivatives of kampferol were represented by: 3-*O*-arabinose and 3-*O*-rhamnose. These peaks had characteristic [M−H]^−^ at *m/z* 417 and *m/z* 431, respectively, and a fragmentation yielding a kaempferol ion at *m/z* 285. Derivatives of quercetin and kaempferol were found in horse chestnut flowers. The structures of these compounds were confirmed by a chemical analysis and spectrophotometric methods (UV, ^1^H-, ^13^C-NMR, ESI-MS) [15]. A range of flavonoid glycosides of quercetin (e.g., quercitrin, rutin, isoquercitrin and quercetin 3-*O*-arabinoside) and the corresponding glycosides of kaemperfol have also been detected in leaf tissues [21]. Results concerning quercetin-3-*O*-rhamnoside are contradictory, with different authors reporting that this compound is present either in the leaves or in the seeds of *A. hippocastanum* [22].

### 2.2. Quantification of Phenolics in Chestnut Horse Leaves

The content of phenolics in white and red chestnut horse leaves which suffered from attack by a leaf mining insect varied significantly, as shown in Table 2. The total content of phenolics in the white chestnut horse leaf extract before the attack by a leaf mining insect in June was 60,086 mg/g dm. 

However, in the infected by a leaf mining insect leaves it was much lower and reached 46,573 mg/g dm in June and 5697 mg/g dm in September. In the extract prepared from red chestnut horse leaves picked in September without leaf mining insects, the total phenolic compounds content was 90,837 mg/g dm and leaves which suffered from the attack by a leaf mining insect at 52,978 mg/g dm, respectively. In the case of horse chestnut, the activity of mining insect caused a decrease of phenolic content. This indicates no activation of any defense mechanism that results in an accumulation of polyphenolic compounds. Petkovšek *et al.* [23] found that infected leaves of apples contained higher concentrations of (−)-epicatechin (1.3 to 3.1 times more) and (+)-catechin (1.2 to 3.1 times more) than healthy leaves. A transient accumulation of some compounds, especially flavan-3-ols, was also detected after pear leaf wounding, thus confirming the role of phenolic compounds in resistance to injury and stress [24]. Treutter *et al.* [14] reported that in pear leaves infected by *Gymnosporangium sabinae* and *Venturia pirina* in the vicinity of the infected plant, the leaf tissue synthesizes more flavan-3-ols. 

However, a higher content of polyphenolic compounds—especially (−)-epicatechin and procyanidins—in the leaves of red-horse chestnut than in the leaves of white-horse chestnut may explain their greater resistance to *C. ohridella* insects. Interesting is the lack of quercetin-3-*O*-rutinoside in the extracts from the leaves of the white horse chestnut compared to red horse chestnut (Table 2, Figure 2). Quercetin-3-*O*-rutinoside is one of the most widely studied flavonol glycosides. The behavioral response of insects to rutin can vary depending on the concentration tested and the age of the insect tested. For example, rutin at concentrations greater than 10^−3^ M deterred the final stadium *Heliothis zea* and *Heliocoverpa armigera* larvae from feeding, but at concentrations lower than 10^−4^ M it stimulated feeding in final stadium larvae. Quercetin-3-*O*-rutinoside also deters the second stadium *H. zea* larvae from feeding [25]. 

### 2.3. Principal Component Analysis (PCA)

**Figure 3:**
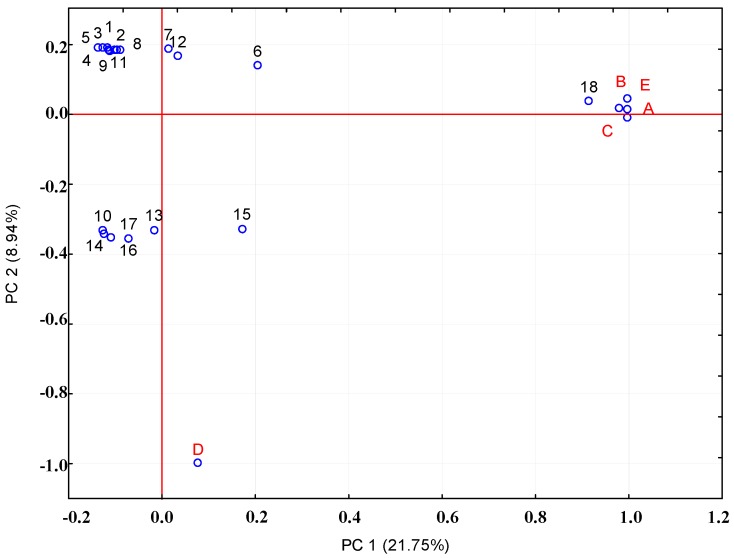
Loading plot for principal component analysis (PCA) of the first two factors. (**A**) white horse chestnut no infected leaves (June); (**B**) white horse chestnut infected leaves (June); (**C**) white horse chestnut infected leaves (September); (**D**) red horse chestnut no infected leaves (September); E- red horse chestnut infected leaves (September)

PCA was conducted to confirm any relationships among the analyzed variables from evaluated white and red horse chestnut samples. After the statistical analysis of all data, the PCA model retained three principal components (PC), which explained 35.52% of the total variability. The score and loading plots of the first two principal components are shown in Figure 3. 

Some defined groups were observed, and relationships among variables were related to the accession. PC1 arranged samples according to some quercetin and keampferol derivatives, revealing a relationship between the content of these compounds and red-horse chestnut non-infected leaves (**D**). PC2 revealed an inverse relationship among the samples of non-infected (**A**) and infected (**B**, **C**) white horse chestnut and infected red horse chestnut (**E**) leaves. Leaves of non-infected (**A**) and infected (**B**, **C**) white-horse chestnut were located as exhibiting a strong relationship with procyanidin polymers [18]. It is very interesting that the rest of compounds flavan-3-ols and phenolic acid **1**–**9** and **12** are located opposite to the sample and other compounds, which means that these substances are not synthesized during infection.

## 3. Experimental

### 3.1. Reagent and Standards

Acetonitrile, formic acid and methanol were purchased from Sigma-Aldrich (Steinheim, Germany). Acetonitrile were purchased from Merck (Darmstadt, Germany). Qercetin-3-*O*-galactoside, quercetin-3-*O*-rhamnoside, quercetin-3-*O*-rutinoside, kaempferol–3-*O*-glucoside, (−)-epicatechin, procyanidin A2 and B2, were purchased from Extrasynthese (Lyon, France). Neochlorogenic acid and caffeic acid were purchased from TRANS MIT GmbH (Giessen, The Netherlands).

### 3.2. Plant Material

The leaves infected with *C. ohridella* and healthy control leaves of white and red horse chestnut were randomly collected from the Garden of Medicinal Plants herbarium at the Medical University in Wroclaw, Poland. White horse chestnut leaves infected and not infected with *C. ohridella* were picked over five time points in June and only infected in September 2013 from the same trees. Red horse chestnut leaves with more resistance and uncontaminated resistance to leaf miner larvae attack were picked at the same time in September. In the course of the measurements, three replicates (10 randomly chosen leaves) from three trees, that is, 30 replicates per time period, were established. The condition of infected leaves was evaluated based on their appearance. After harvest the leaves were cut and directly frozen in liquid nitrogen, and freeze-dried (24 h; Christ Alpha 1–4 LSC; Martin Christ GmbH, Osterode am Harz, Germany). The homogeneous powders were obtained by crushing the dried tissues using a closed laboratory mill to avoid hydration (IKA 11A; Staufen, Germany). Powders were kept in a freezer (−80 °C) until extract preparation.

### 3.3. Extraction Procedure

The powder samples (1 g) were extracted with methanol acidified with 1% acetic acid (25 mL). The extraction was performed twice by incubation for 20 min under sonication and with occasional shaking. Next, the slurry was centrifuged at 19,000× *g* for 10 min and the supernatant was filtered through a 0.25 µm membrane, and used for analysis. The identification and content of polyphenols in individual extracts was determined by means of the liquid chromatography (UPLC-PDA and LC-/MS) method.

### 3.4. Identification of Polyphenols by the Liquid Chromatography-Mass Spectrometry (LC-MS) Method

Identification of polyphenols of horse chestnut leaf extracts was carried out using an ACQUITY Ultra Performance LC^TM^ system with a mass detector G2 QTof Micro mass spectrometer equipped with an electrospray ionization (ESI) sources operating in negative modes (UPLC^TM^; Waters Corporation; Milford, MA, USA). Separations of individual polyphenols were carried out using a UPLC BEH C18 column (1.7 μm, 2.1 × 100 mm, Waters Corporation) at 30 °C. The elution solvents were aqueous 4.5% formic acid (A) and 100% acetonitrile (B). Samples (10 μL) were eluted according to the linear gradient described by Oszmiański *et al.* [26]. Analysis was carried out using full scan, data-dependent MS scanning from *m/z* 100 to 1500. The mass tolerance was 0.001 Dalton and the resolution was 5.000. Leucine enkephalin was used as the internal reference compound during ESI-MS accurate mass experiments and was permanently introduced via the LockSpray channel using a Hamilton pump. The Lock Mass Correction was +/– 1.000 for Mass Window. All TOF-MS-chromatograms are displayed as Base Peak Intensity (BPI) chromatograms and scaled to 12,400 counts per second (cps) (=100%). The effluent was led directly to an electrospray source with a source block temperature of 130 °C, desolvation temperature of 350 °C, capillary voltage of 2.5 kV and cone voltage of 30 V. Nitrogen was used as desolvation gas flow rate 300 L·h^−1^.

The characterization of the single components was carried out via their retention times and their accurate molecular masses. Each compound was optimized to its estimated molecular mass [M−H]^−^ in the negative mode before and after fragmentation. The data obtained from LC/MS were subsequently entered into the MassLynx 4.0 ChromaLynxTM Application Manager software. Based on these data, the software is able to scan different samples for the characterized substances.

The runs were monitored at the following wavelengths: (‒)-Epicatechin and procyanidin at 280 nm, hydroxycinnamates at 320 nm, and flavonol glycosides at 360 nm. Retention times (R_t_) and spectra were compared with those of pure standards. Calibration curves at concentrations ranging from 0.05 to 5 mg/mL (r^2^ ≤ 0.9998) were made from (−)-epicatechin, procyanidin A2, procyanidin B2, neochlorogenic acid, caffeic acid, quercetin-3-*O*-galactoside, quercetin-3-*O*-rutinoside, kaempferol-3*-O*-glucoside as standards. All incubations were done in triplicate. The results was expressed as milligrams per g dry matter (dm).

### 3.5. Analysis of Proanthocyanidins by Phloroglucinolysis Method

Direct phloroglucinolysis of freeze-dried horse chestnut leaves was performed as described previously by Wojdyło *et al.* [27]. Portions (0.05 g) of powder were precisely measured into 2 mL Eppendorf vials, then methanolic solution (0.8 mL) of phloroglucinol (75 g/L) and ascorbic acid (15 g/L) was added. After the addition of methanolic HCl (0.4 mL, 0.3 mol/L), the vials were closed and incubated for 30 min at 50 °C with continuous vortexing using a thermo shaker (TS-100; BIOSAN, Riga, Lithuania). The reaction was stopped by placing the vials in an ice bath by drawing 0.5 mL of the reaction medium and diluting with 0.5 mL of 0.2 mol/L sodium acetate buffer. Next the vials were cooled in ice water and centrifuged immediately at 20,000× *g* for 10 min at 4 °C. The analytical column was kept at 15 °C by column oven, whereas the samples were kept at 4 °C. 

The analysis of polymeric procyanidins compounds was carried out on an Acquity UPLC system (Waters Corp.) consisting of a binary solvent manager, and fluorescence detector (FL). Empower 3 software was used for chromatographic data collection and integration of chromatograms. A partial loop injection mode with a needle overfill was set up, enabling 5 μL injection volumes when a 10 μL injection loop was used. Acetonitrile (100%) was used as a strong wash solvent and acetonitrile–water (10%) as a weak wash solvent. The analytical column BEH Shield C18 (2.1 mm × 50 mm; 1.7 μm) was kept at 15 °C by column oven, whereas the samples were kept at 4 °C. The flow rate was 0.45 mL/min. The mobile phase was composed of solvent A (2.5% acetic acid) and solvent B (acetonitrile). Elution was as follows: 0–0.6 min, isocratic 2% B; 0.6–2.17 min, linear gradient from 2% to 3% B; 2.17–3.22 min, linear gradient from 3% to 10% B; 3.22–5.00 min, linear gradient from 10% to 15% B; 5.00–6.00 min, column washing; and reconditioning for 1.50 min. The fluorescence detection was recorded at an excitation wavelength of 278 nm and an emission wavelength of 360 nm. The calibration curves, which were based on peak area, were established using (+)-catechin, (−)-epicatechin, and procyanidin B1 after phloroglucinol reaction as (+)-catechin- and (−)-epicatechin—phloroglucinol adduct standards. The average degree of polymerization was calculated as the molar ratio of all the flavan-3-ol units (phloroglucinol adducts + terminal units) to (−)-epicatechin and (+)-catechin, which correspond to terminal units. All incubations were done in triplicate. The results was expressed as milligrams per g dm.

### 3.6. Principal Component Analysis

Principal component analysis (PCA) was performed using STATISTICA v. 10 (Kraków, Poland) on mean values of three samples and 31 variables. The PCA is a multilinear descriptive method.

## 4. Conclusions

Normally, plant phenolics are secondary metabolites involved in the defense mechanisms of plants against fungal pathogens. In our research we compared infected and non-infected leaves of white and red horse chestnut leaves. We observed a significantly decreased concentration of some phenolic compounds in leaves after infection, especially flavan-3-ols. Additionally a higher content of polyphenolic compounds, especially (−)-epicatechin and procyanidins in leaves of red-flowering than in white-flowering horse chestnut may explain their greater resistance to *C. ohridella* insects. Further investigations and observations are however needed, that would enable identifying some correlations between pathogen attack and decreased content of polyphenols in the whole vegetative season of horse chestnut. Based on the data obtained, it may be speculated that the activity of *C. ohridella* makes that the vital functions of leaves die out and polyphenolic compounds are incapable of playing the role of phytoalexins. 

## References

[1] Simova-Tosic D., Filev S. (1985). Contribution to the horse chestnut miner. Zast. Bilja.

[2] Deschka G., Dimic N. (1986). *Cameraria ohridella* n. sp. aus Mazedonien, Jugoslawien (Lepidoptera: Lithocolletidae). Acta Entomol. Jugosl..

[3] Guichard S., Augustin S. (2002). Acute spread in France of an invasive pest, the horse chestnut leaf-miner *Cameraria ohridella* (Lepidoptera: Garcillariidae). J. Pest Sci..

[4] Baraniak E., Walczak U., Zduniak P. (2005). Appearance and migration of the horse-chestnut leafminer Cameraria ohridella in relation to city size and leaf-raking, using the example of two cities in Western Poland. J. Pest Sci..

[5] Pschorn-Walcher H. (1994). Field biology of the introduced horse-chestnut leafminer *Cameraria ohridella* Deschka et Dimič (*Lepidoptera: Gracillariidae*) in theVienna Woods. Linzer Biol. Beitr..

[6] Percival G.C., Barrow I., Noviss K., Keary I., Pennington P. (2011). The impact of horse chestnut leaf miner (*Cameraria ohridella* Deschka and Dimic) on vitality, growth and reproduction of Aesculus hippocastanum L.. Urban For. Urban Green..

[7] Nardini A., Raimondo F., Scimone M., Salleo S. (2004). Impact of the leaf miner *Cameraria ohridella* on whole-plant photosynthetic productivity of *Aesculus hippocastanum*: Insights from a model. Trees.

[8] Raimondo F., Ghirardella L.A., Nardini A., Salleo S. (2003). Impact of the leaf miner Cameraria ohridella on photosynthesis, water relations and hydraulics of *Aesculus hippocastanum* leaves. Trees.

[9] Thalmann C., Freise J., Heitland W., Bacher S. (2003). Effects of defoliation by horse chestnut leaf miner *Cameraria ohridella* on reproduction in *Aesculus hippocastanum*. Trees.

[10] Kukuła-Młynarczyk A., Hurej M., Jackowski J. (2006). Development of horse chestnut leafminer (*Cameraria ohridella* Deschka and Dimić) on red horse chestnut. J. Plant Prot. Res..

[11] Dzięgielewska M., Kaup G. (2007). Occurrence of chesnut leaf miner (*Cameraria ohridella*) on red horse chustnut (*Aesculus* x *carnea*) in Szczecin. Prog. Plant Prot..

[12] Bennet R.N., Wallsgrove R.M. (1994). Secondary metabolites in plant defense mechanisms. New Phytol..

[13] Michalek S., Mayr U., Treutter D., Lux-Endrich A., Gutmann M., Feucht W., Geibel M. (1999). Role of flavan-3-ols in resistance of apple trees to *Venturia inaequalis*. Acta Hortic..

[14] Treutter D., Feucht W. (1990). Accumulation of flavan-3-ols in fungus-infected leaves of Rosaceae. Z. Pflanzeik. Pflanzen..

[15] Dudek-Makuch M., Matławska I. (2011). Flavonoids from the flowers of *Aesculus hippocastanum*. Acta Pol. Pharm..

[16] Kapusta I., Janda B., Szajwaj B. (2007). Flavonoids in horse chestnut (*Aesculus hippocastanum*) seeds and powdered waste water byproducts. J. Agric. Food Chem..

[17] Hübner G., Wray V., Nahrstedt A. (1999). Flavonol oligosaccharides from the seeds of Aesculus hippocastanum. Planta Med..

[18] Broda B. (2002). Zarys botaniki farmaceutycznej.

[19] Tomczyk A., Kropczyńska-Linkiewicz D., Ptak A. (2008). Use of extracts fromthe leaves of white and red horse-chestnut to the studies on the plant acceptance by horse-chestnut leafminer (*Cameraria ohridella*). Prog. Plant Prot..

[20] Bruneton J. (1996). Pharmacognosy, Phytochemistry, Medicinal Plants.

[21] Bisset N.G. (1994). Herbal Drugs and Phytopharmaceuticals—A Handbook for Practice on a Scientific Basis.

[22] Bombardelli E., Morazzoni P., Griffini A. (1996). *Aesculus hippocastanum* L.. Fitoterapia.

[23] Petkovšek M.M., Stampar F., Veberic R. (2008). Increased phenolic content in apple leaves infected with the apple scab pathogen. J. Plant Pathol..

[24] Andreotti C., Costa G., Treutter D. (2006). Composition of phenolic compounds in pear leaves as affected by genetics, ontogenesis and the environment. Sci. Hortic.-Amsterdam.

[25] Isman M.B., Duffey S.S. (1982). Toxicity of tomato phenolic compounds to the fruit form *Heliothis zea*. Entomol. Exp. App..

[26] Oszmiański J., Kolniak-Ostek J., Wojdyło A. (2013). Application of Ultra Performance Liquid Chromatography-Photodiode Detector-Quadrupole/Time of Flight-Mass Spectrometry (UPLC-PDA-Q/TOF-MS) method for the characterization of phenolic compounds of *Lepidium sativum* L. sprouts. Eur. Food Res. Technol..

[27] Wojdyło A., Oszmiański J., Bielicki P. (2013). Polyphenolic composition, antioxidant activity, and polyphenol oxidase (PPO) activity of quince (*Cydonia oblonga* Miller) varieties.. J. Agric. Food Chem..

